# Tsc1 expression by dendritic cells is required to preserve T-cell homeostasis and response

**DOI:** 10.1038/cddis.2016.487

**Published:** 2017-01-12

**Authors:** Yuechen Luo, Wenwen Li, Gang Yu, Juan Yu, Ling Han, Ting Xue, Zhina Sun, Song Chen, Chunming Fang, Chunxiao Zhao, Qing Niu, Fei Yang, Zhongchao Han, Tao Cheng, Yun Zeng, Fang Liao, Guogang Xu, Xiaoming Feng

**Affiliations:** 1 State Key Laboratory of Experimental Hematology, Institute of Hematology and Hospital of Blood Diseases, Chinese Academy of Medical Sciences & Peking Union Medical College, Tianjin 300020, China; 2 Nanlou Respiratory Department, Chinese PLA General Hospital, Beijing 100853, China; 3 Department of Respiratory Medicine, The Second affiliated hospital of Nanchang University, Nanchang, Jiangxi 33000, China; 4 Department of Medical Microbiology, Tongji Medical College, Huazhong University of Science and Technology, Wuhan 430030, China; 5 Department of Hematology, First Affiliated Hosptial of Kunming Medical University, Kunming 650032, China

## Abstract

Dendritic cells (DCs) are pivotal to the induction of adaptive T-cell immune responses. Recent evidence highlights a critical role of tuberous sclerosis complex 1 (Tsc1), a primarily upstream negative regulator of mammalian target of rapamycin (mTOR), in DC development, but whether and how Tsc1 directly regulate mature DC function *in vivo* remains elusive. Here we show that selective disruption of Tsc1 in DCs results in a lymphoproliferative disorder with the spontaneous activation of T cells. Tsc1 deficiency results in the activation of mTORC1-PPAR*γ* pathway, which leads to the upregulation of neuropilin-1 (Nrp1) expression on DCs to stimulate naive T-cell proliferation. However, Tsc1-deficient DCs have defects in the ability to induce antigen-specific T-cell responses *in vitro* and *in vivo* owing to impaired survival during antigen transportation and presentation. Indeed, Tsc1 promotes DC survival through restraining independent mTORC1 and ROS-Bim pathways. Our study identifies Tsc1 as a crucial signaling checkpoint in DCs essential for preserving T-cell homeostasis and response.

Dendritic cells (DCs) are specialized sentinels that induce adaptive immune responses according to environmental stimuli.^1, 2^ Under steady-state conditions, DCs contribute to immunological tolerance against self-antigens.^3^ During overt immunization or infection, foreign antigens activate DCs to upregulate the expression of major histocompatibility complex (MHC) molecules, co-stimulatory molecules and cytokines to trigger adaptive T-cell responses.^4, 5^ How DCs shape an efficient immune response to peripheral cues while avoiding immune activation under steady-state conditions remains incompletely understood.

Mammalian target of rapamycin (mTOR) is a central integrator of immune responses, and its activity is repressed by the upstream tuberous sclerosis complex 1 (Tsc1)–Tsc2 complex.^6, 7^ Several studies indicated that mTOR signaling was a particularly critical regulator of DC differentiation, maturation and function.^8, 9, 10, 11, 12, 13^ Three recent studies investigated the roles of Tsc1 in DC development and activation. Pan *et al.*^14^ reported that Tsc1 increased IRF4/CIITA/MHC II expression in bone marrow (BM)-derived DCs to enhance OT-II T-cell priming without affecting DC development in response to GM-CSF. But this study was limited by its basis in the *in vitro*-cultured BM-derived DC system, which may be different from DCs *in vivo*.^2^ Wang *et al.*^15^ demonstrated that Tsc1-deficient DC precursors were inclined to differentiate into macrophages and neutrophils, exhibited impaired survival and IL-12 production and therefore arrested DC-mediated Th1 immune responses, although it had increased expression of CD40, CD80 and CD86. This study suggested that Tsc1 ablation impaired DC development and consequently attenuated DC T-cell response. Further, Sathaliyawala *et al.*^9^ identified that Tsc1 loss did not affect DC terminal differentiation or maintenance. These results collectively support an important but complex role of Tsc1 in DC development. However, the precise role and underlying molecular mechanism of Tsc1 in mature DC function *in vivo* are still not well defined.

Here we investigate the direct role of Tsc1 in mature DC function and the potential molecular basis using a mouse line with Tsc1 specifically deleted in CD11c^+^ DCs (*CD11c*^Cre^*Tsc1*^f/f^ mice). Distinct from the results observed in BM-derived DCs,^14, 15^ we did not detect significantly altered expression of MHC II, co-stimulatory molecules CD40, CD80 and CD86, cytokine IL-12 or abnormal differentiation in DCs from secondary lymphoid organs in *CD11c*^Cre^*Tsc1*^f/f^ mice. Instead, we found that specific depletion of Tsc1 in DCs resulted in the development of splenomegaly and lymphadenopathy, increased serum immunoglobulin levels and body weight loss in mice, which were characteristics of spontaneous autoimmunity. The mTORC1–PPAR*γ* axis-dependent upregulation of neuropilin 1 (Nrp1) in Tsc1-deficient DCs drove naive T-cell proliferation. In contrast, Tsc1-deficient DCs showed a defective ability to induce antigen-specific responses *in vitro* and *in vivo* as a result of severely reduced number of DCs and hesitated to drive Th2 and Th17 immune response in asthma model. Mechanistically, mTORC1- and ROS-Bim-induced excessive apoptosis of Tsc1-deficient DCs during antigen transportation and presentation then prevented the efficient priming of antigen-specific T-cell responses. Thus our data define Tsc1 as a critical regulator in mature DCs to ensure T-cell homeostasis and immune response.

## Results

### Tsc1 in DCs prevents the development of lymphoproliferative disorder

**Figure 1:**
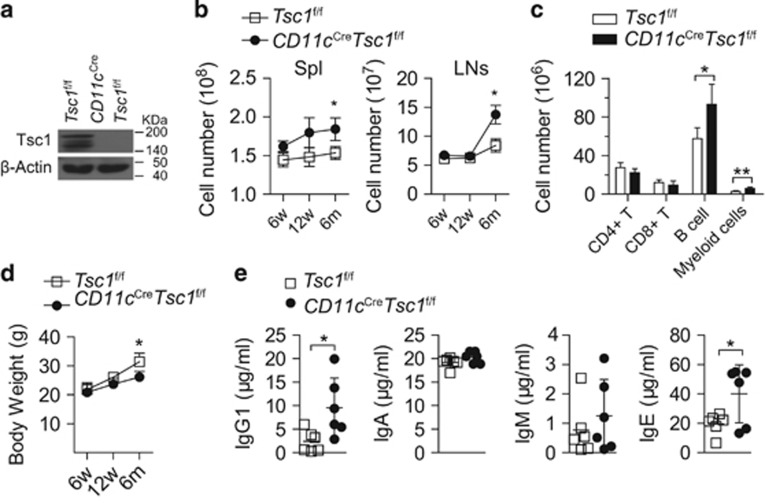
Tsc1 in DCs prevents the development of lymphoproliferative disorder. (**a**) Tsc1 protein was depleted in splenic DCs from *CD11c*^Cre^*Tsc1*^f/f^ mice, determined by western blotting. (**b**) Total cell numbers in the spleen (Spl) and lymph nodes (LNs) from mice at different ages (*n*=4). (**c**) The numbers of different cell populations in the spleen and lymph nodes of 6-month-old mice (*n*=4). Myeloid cells included macrophages, neutrophils and monocytes. (**d**) Body weight of *Tsc1*^f/f^ and *CD11c*^Cre^*Tsc1*^f/f^ male mice of different ages (*n*=6). (**e**) Concentrations of serum IgG1, IgA, IgM and IgE in 6-month-old mice (*n*=6). All mice analyzed were 6 week old, unless otherwise specified. *P<0.05, **P<0.01; error bars represent S.D.; all data are representative of at least three independent experiments

To determine whether Tsc1 in DCs regulates T-cell homeostasis and response *in vivo*, we generated a mouse line with Tsc1 conditionally depleted in DCs by crossing *Tsc1*^f/f^ mice (mice with *loxP*-flanked alleles of the gene encoding mouse Tsc1^15^) with *CD11c*^Cre^ mice (mice with transgenic expression of Cre recombinase under the control of the CD11c promoter^16^). Tsc1 protein was ablated in splenic DCs from *CD11c*^Cre^*Tsc1*^f/f^ mice (Figure 1a). Despite being outwardly healthy, *CD11c*^Cre^*Tsc1*^f/f^ mice developed a lymphoproliferative disorder characterized by considerable splenomegaly and lymphadenopathy with increased total cell numbers with age (Figure 1b). Further analysis revealed that a population expansion of B cells and myeloid cells accounted for the increased cellularity of secondary lymphoid organs in elder *CD11c*^Cre^*Tsc1*^f/f^ mice (Figure 1c). We examined the body weight and found that the elder *CD11c*^Cre^*Tsc1*^f/f^ mice displayed lower body weight than *Tsc1*^f/f^ mice (wild-type (WT) mice) (Figure 1d). Furthermore, serum levels of IgG1 and IgE in *CD11c*^Cre^*Tsc1*^f/f^ mice were higher than in WT mice (Figure 1e), indicating a role of Tsc1 in autoimmune disorders. However, we did not observe alterations in histopathology (data not shown) and specific markers of autoimmune disease in *CD11c*^Cre^*Tsc1*^f/f^ mice (Supplementary Figure S1). These findings indicate a role of Tsc1 in DCs for preserving immune homeostasis in steady-state conditions.

### Tsc1 in DCs prevents spontaneous T-cell activation *in vivo*

**Figure 2:**
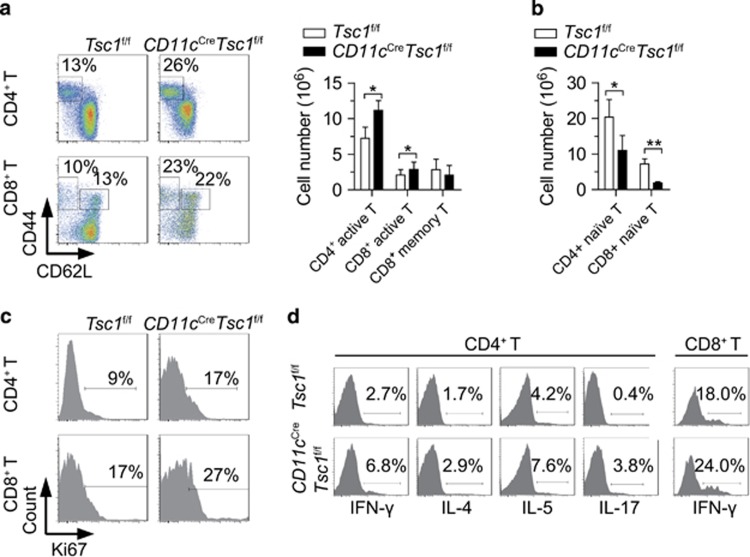
Tsc1 in DCs prevents spontaneous T-cell activation *in vivo*. (**a**) Expression of CD44 and CD62L on splenic CD4^+^Foxp3^−^ and CD8^+^Foxp3^−^ T cells. (**b**) Absolute number of CD44^−^CD62L^+^ naive T cells in the spleen (*n*=4). (**c**) Ki67 staining of splenic T cells. (**d**) Intracellular staining of cytokines in splenic CD4^+^Foxp3^−^ and CD8^+^Foxp3^−^ T cells, stimulated with phorbol myristate acetate and ionomycin for 4 h. All mice analyzed were 6 week old. *P<0.05, **P<0.01; error bars represent S.D.; all data are representative of at least three independent experiments

The major function of DCs is controlling the homeostasis and function of T cells. We found a significantly higher frequency and number of CD4^+^ and CD8^+^ T cells that displayed the CD44^high^ CD62L^low^ activated/memory phenotype in *CD11c*^Cre^*Tsc1*^f/f^ mice (Figure 2a). In contrast, the naive CD4^+^ and CD8^+^ T cells were progressively lost with age (Figure 2b). T cells from *CD11c*^Cre^*Tsc1*^f/f^ mice also exhibited increased proliferation and higher production of the cytokines IFN-*γ*, IL-4, IL-5 and IL-17 (Figures 2c and d). These observations indicate that Tsc1-deficient DCs delivers strong signals to induce aberrant activation and proliferation of naive T cells without foreign antigen challenge.

### Tsc1 represses Nrp1 in DCs to prevent naive T-cell proliferation

**Figure 3:**
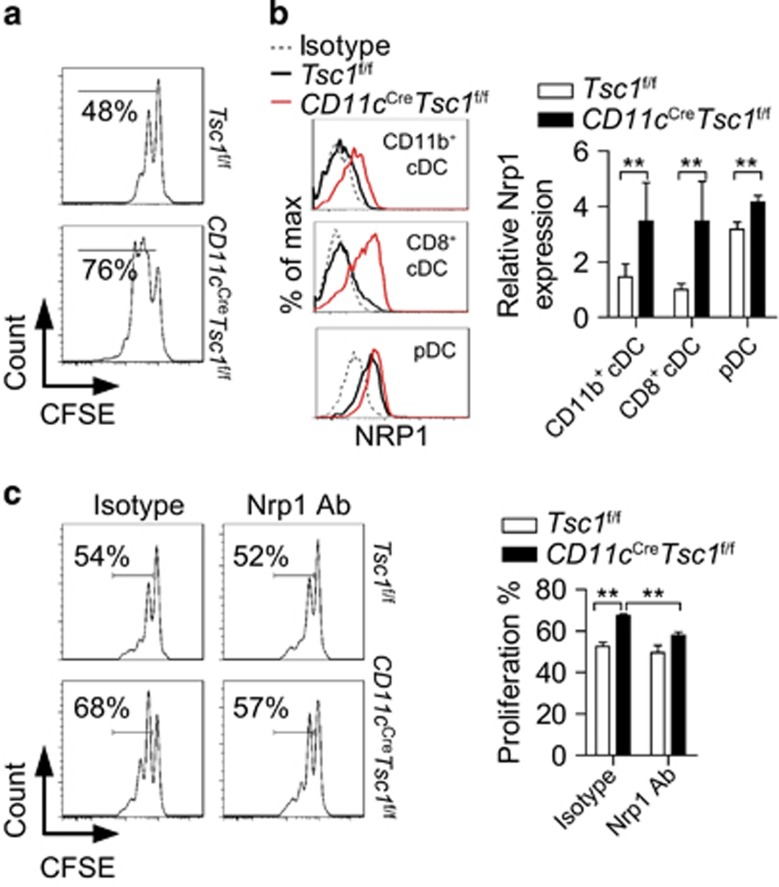
Tsc1 represses Nrp1 in DCs to prevent antigen-independent naive T-cell proliferation. (**a**) Proliferation of CFSE-labeled CD4^+^CD44^−^CD62L^+^ naive T cells, after being co-cultured with splenic DCs for 72 h. IL-7 (100 ng/ml) was added to the culture solution. (**b**) Expression of Nrp1 on splenic cDCs and pDCs (*n*=3). (**c**) Proliferation of CFSE-labeled CD4^+^CD44^−^CD62L^+^ naive T cells after being co-cultured with splenic DCs for 72 h and treated with Nrp1-Ab or isotype (*n*=3). IL-7 (100 ng/ml) was added to the culture solution. All mice analyzed were 6 week old. **P<0.01; error bars represent S.D.; all data are representative of at least two independent experiments

To investigate whether Tsc1-deficient DCs directly induce T-cell activation, we used the *in vitro* DC-naive T-cell co-culture system and found that Tsc1-deficient DCs induced more proliferation of naive T cells, in the absence of foreign antigen (Figure 3a).

DC activation and functional maturation have always been associated with increased expression of MHC class II, co-stimulatory molecules and cytokines.^5^ We found that Tsc1-deficient DCs were bigger in cell size and had slightly increased expression of CD80 but not of MHC or other common co-stimulatory molecules (Supplementary Figure S2a). Cytokine production was comparable between WT and Tsc1-deficient DCs (Supplementary Figure S2b). These results suggest that loss of Tsc1 does not lead to overt activation of DCs. As the number of DCs was decreased in secondary lymphoid organs, elevated naive T-cell proliferation could not be attributed to decreased numbers of DCs (Supplementary Figure S2c).

Nrp1 is a membrane molecule that is known to be essential for driving naive T-cell proliferation by DCs.^17^ We hypothesized that the increased proliferation of naive T cells is partly dependent upon Nrp1. Indeed, although barely expressed in WT DCs, a large amount of Nrp1 was expressed in Tsc1-deficient DCs (Figure 3b). We further explored whether the increased Nrp1 expression contributed to the naive T-cell proliferation. Blocking Nrp1, with a neutralizing antibody, partly dampened the enhanced priming of naive CD4^+^ T cells by Tsc1-deficient DCs (Figure 3c). Thus the aberrant upregulation of Nrp1 in Tsc1-deficient DCs accounts, at least partially, for the T-cell activation/proliferative phenotype in *CD11c*^Cre^*Tsc1*^f/f^ mice.

### Tsc1 represses Nrp1 expression through inhibiting mTORC1-PPAR-*γ* pathway

**Figure 4:**
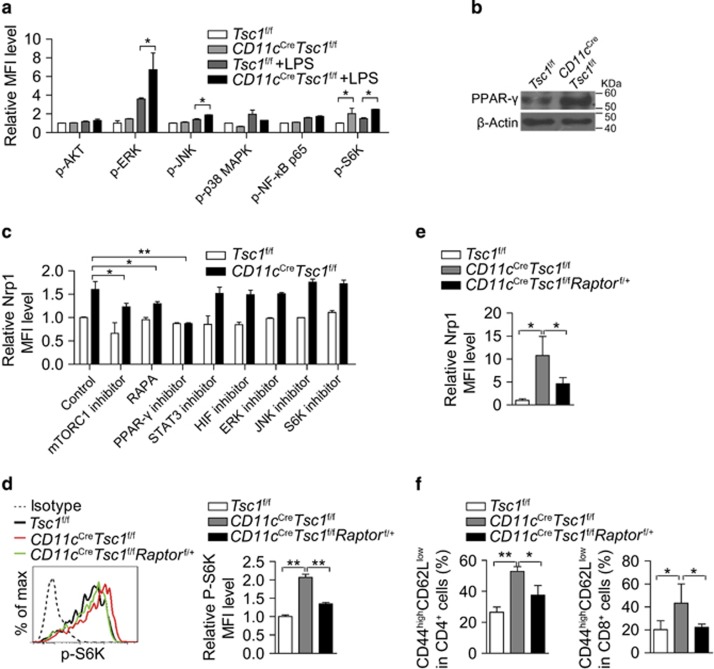
Tsc1 represses Nrp1 expression through inhibition of mTORC1-PPAR-*γ* pathway. (**a**) Intracellular phosphorylated Akt (Ser 473), ERK1/2 (Thr 202/204), JNK (Tyr 185), p38 MAPK (Thr 180/Tyr 182), NF-*κ*B p65 (Ser 536) and S6K (Ser 235/Ser 236) in splenic DCs from *Tsc1*^f/f^ and *CD11c*^Cre^*Tsc1*^f/f^ mice, treated with or without LPS (*n*=3). (**b**) PPAR-*γ* protein was increased in splenic DCs from *CD11c*^Cre^*Tsc1*^f/f^ mice, determined by western blotting. (**c**) Nrp1 expression levels on splenic DCs after being treated with RAPA (50 ng/ml), inhibitors of mTORC1 (everolimus, 10 *μ*M), PPAR-*γ* (1 nM), STAT3 (5 *μ*M), HIF (10 *μ*M), ERK (1 *μ*M), JNK (10 nM) or S6K (50 nM) for 16 h (*n*=3). (**d**) Intracellular phosphorylated S6K (Ser 235/Ser 236) in splenic DCs (*n*=3). (**e**) Expression of Nrp1 on splenic DCs (*n*=3). (**f**) Percentages of CD44^high^CD62L^low^ effector/memory cells among CD4^+^Foxp3^−^ and CD8^+^Foxp3^−^ T cells from the indicated mice (*n*=6). All mice analyzed were 6 week old. *P<0.05, **P<0.01; error bars represent S.D.; all data are representative of at least three independent experiments

We next explored the signaling pathway alterations in Tsc1-deficient DCs. Owing to the rarity of the DCs *in vivo*, we used phospho-flow cytometry to examine the activation of the major signaling pathways, including AKT, Erk, JNK, p38MAPK, NF-*κ*B, S6K and PPAR-*γ*, in Tsc1-deficient DCs. We found that the mTORC2 substrate Akt (Ser473),^18^ p38MAPK and NF-*κ*B activation were barely altered, and Erk and JNK activation were slightly increased, whereas S6K and PPAR-*γ* activation were significantly increased in Tsc1-deificent DCs compared with that in WT cells (Figures 4a and b). Although Myc has been reported to be elevated in Tsc1-deficient BM-derived DCs,^15^ we did not find altered expression of Myc in splenic Tsc1-deficient DCs (data not shown). We utilized a panel of chemical inhibitors to determine which signaling alteration caused the upregulation of Nrp1 in Tsc1-deficient DCs. Both *in vivo* administration of the mTORC1 inhibitor rapamycin (RAPA) and *in vitro* treatment with the mTORC1 inhibitor everolimus^19^ downregulated the expression of Nrp1 in DCs (Figure 4c). These results suggest that mTORC1-activated transcription factors might regulate Nrp1 gene expression. The known mTORC1-regulated transcription factors include PPAR-*γ*, STAT3, and HIF.^6^ Indeed, inhibition of PPAR-*γ*, but not STAT3 and HIF, reduced the expression of Nrp1 in Tsc1-deficient DCs.

In addition to mTORC1, Tsc1 might also act through other signaling pathways.^20^ To precisely determine the effects of mTORC1 hyperactivation on the biological function of Tsc1-deficient DCs, we generated *CD11c*^Cre^*Tsc1*^f/f^*Raptor*^f/+^ mice to deplete *Tsc1* and one allele of *Raptor*, which is an obligatory component of mTORC1,^6^ in which the hyperactivation of mTORC1 was diminished (Figure 4d). The increased expression of Nrp1 in DCs and the T-cell activation phenotype were largely rescued in *CD11c*^Cre^*Tsc1*^f/f^*Rapto*r^f/+^ mice (Figures 4e and f). Taken together, Tsc1 represses Nrp1 expression in DCs through suppressing the mTORC1-PPAR*γ* signaling pathway to prevent spontaneous T-cell activation in steady-state conditions.

### DCs need Tsc1 to promote antigen-specific T-cell responses

**Figure 5:**
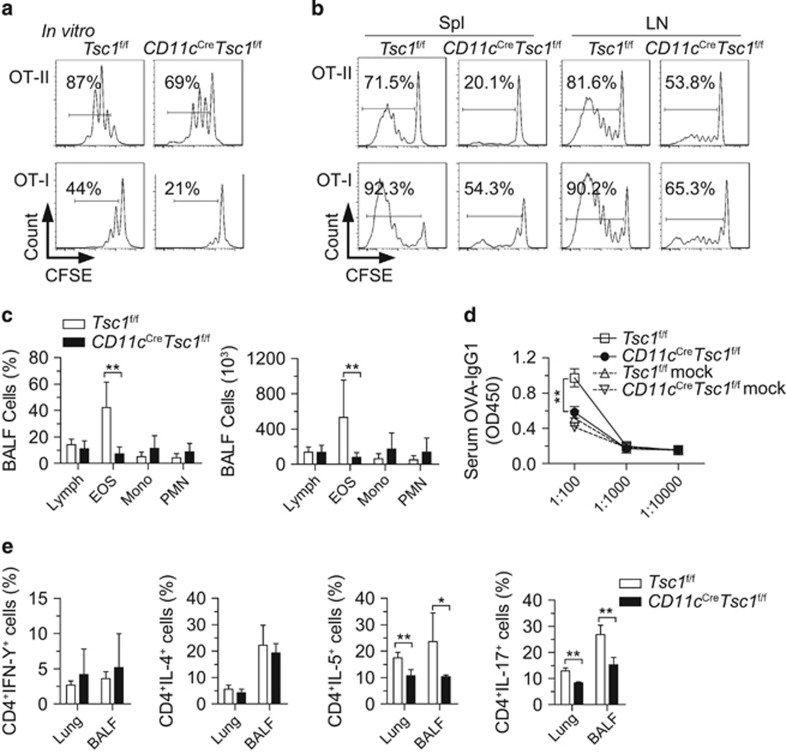
DCs need Tsc1 to promote antigen-specific T-cell responses. (**a**) Proliferation of CFSE-labeled purified OT-II/OT-I T cells, after being co-cultured with splenic DCs for 72 h, which were treated with OVA_323-339_/OVA_257-264_ for 2 h before co-culture. (**b**) CFSE-labeled OT-II/OT-I cells (CD45.1) were i.v. injected into WT or *CD11c*^Cre^*Tsc1*^f/f^ mice (CD45.2). After 1 day, recipient mice were challenged by subcutaneous injection of 10 *μ*g OVA protein with 100 *μ*l complete Freund's adjuvant in each flank, and 3 days later, the spleen was removed and analyzed by flow cytometry. (**c**) The cell percentages and numbers of bronchoalveolar lavage fluid (BALF) in the asthma model (*n*=6). (**d**) Concentration of serum OVA-IgG1 in the asthma model (*n*=6). (**e**) Intracellular staining of cytokines in CD4^+^Foxp3^−^ and CD8^+^Foxp3^−^ T cells in the lung and BALF from WT and *CD11c*^Cre^ *Tsc1*^f/f^ mice, stimulated with phorbol myristate acetate and ionomycin for 4 h. All mice analyzed were 6 week old. *P<0.05, **P<0.01; error bars represent S.D.; all data are representative of at least three independent experiments

To test whether Tsc1 in DCs is important for foreign antigen-driven T-cell immune responses, we first co-cultured WT or Tsc1-deficient DCs with CD4^+^ T cells from OT-II mice or CD8^+^ T from OT-I mice. Surprisingly, splenic Tsc1-deficient DCs showed a considerable defect in the ability to drive antigen-specific T-cell proliferation *in vitro* (Figure 5a). *In vivo*, Tsc1-deficient DCs also had an apparent impairment in inducing proliferation of OT-II and OT-I T cells, which were adoptively transferred into *CD11c*^Cre^*Tsc1*^f/f^ mice immunized with ovalbumin (OVA) (Figure 5b).

We further tested the ability of DCs to mount antigen-specific immune responses to OVA in an asthma model. Here we showed that *CD11c*^Cre^*Tsc1*^f/f^ mice had a profound impairment in adaptive T-cell immunity in this model (Figure 5c). Systemic antibody production and Th2 and Th17 responses were significantly reduced in *CD11c*^Cre^*Tsc1*^f/f^ mice (Figures 5d and e). Together, these findings suggest that *CD11c*^Cre^*Tsc1*^f/f^ mice have a profound defect in eliciting antigen-specific T-cell responses both *in vitro* and *in vivo*.

### Tsc1 maintains sufficient numbers of antigen-pulsed DCs in foreign antigen-specific T-cell responses

**Figure 6:**
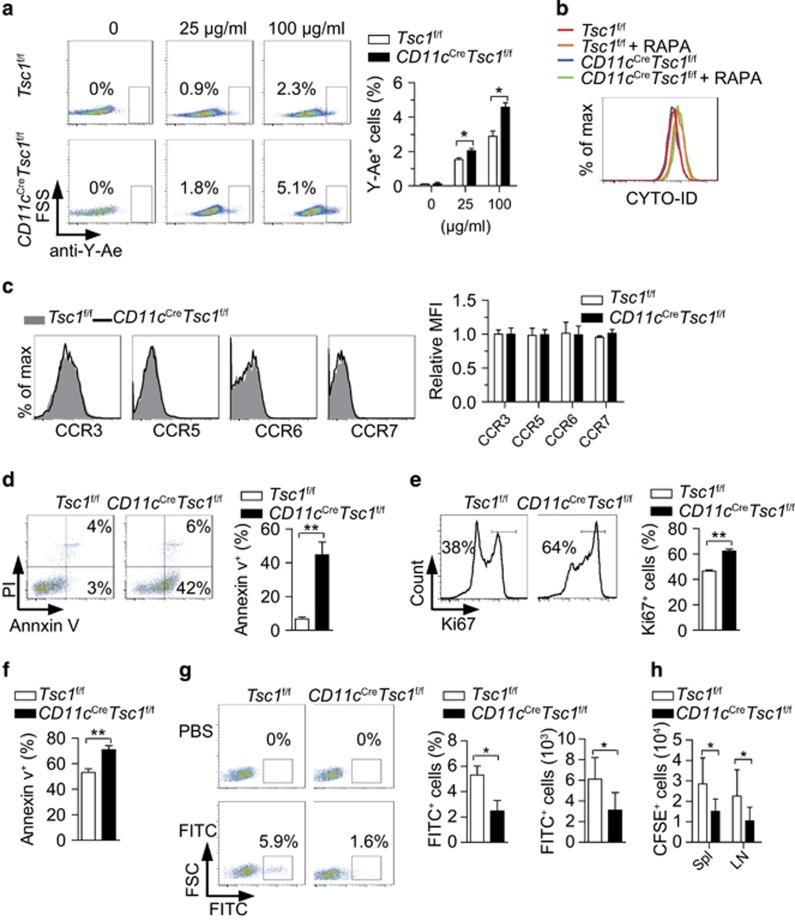
Tsc1 maintains sufficient numbers of antigen-pulsed DCs in antigen-specific T-cell responses. (**a**) Purified DCs were administered Eα antigen for 6 h, and the efficiency of the formation of peptide–MHC class II complexes was determined by staining with the Y-Ae antibody (*n*=3). (**b**) Autophagy detection of splenic DCs, treated with or without RAPA (50 ng/ml) for 16 h. (**c**) Expression of CCR3, CCR5, CCR6 and CCR7 on splenic DCs. (**d**) Annexin V and PI staining of splenic DCs (*n*=3). (**e**) Ki67 staining of splenic DCs (*n*=3). (**f**) Apoptosis of splenic DCs in DC-OT-II co-culture system, after being co-cultured for 3 days(*n*=5). (**g**) The percentage of FITC^+^ cells in draining LNs. The skin of the scapular region of WT and *CD11c*^Cre^*Tsc1*^f/f^ mice was painted with 20 *μ*l of 1% FITC. After 48 h, draining LNs were collected and analyzed. (**h**) Numbers of CFSE^+^ cells in the spleen and LNs from recipients B6.SJL (CD45.1), 3 days after being transferred with CFSE-stained 1 × 10^6^ WT or Tsc1-deficient DCs by i.v. (*n*=5). *P<0.05, **P<0.01; error bars represent S.D.; all data are representative of at least three independent experiments

A previous study demonstrated Tsc1-deficient BM-derived DCs have an impaired capacity to induce an antigen-specific T-cell response owing to diminished IRF4/CIITA/MHCII expression.^14^ Another work indicated that induced deletion of Tsc1 in the early stage of hematopoietic cells caused abnormal development, which resulted in an impaired Th1 response in the mice.^15^ However, in our *CD11c*^Cre^*Tsc1*^f/f^ mice system, we did not observe altered expression of MHC in Tsc1-deficient DCs (Supplementary Figure S1a) or abnormal accumulation of F4/80 macrophage or Ly6G neutrophil differentiation *in vivo* (data not shown). In addition, Tsc1-deficient DCs did not display reduced expression of co-stimulatory molecules (Supplementary Figure S1a) or decreased production of T helper cell cytokines (Supplementary Figure S1b). To examine the ability of DC antigen processing, a 33 amino-acid peptide derived from the histocompatibility factor H2-Eα was administered, and then the specific Y-Ae antibody was used to detect the presented I-A^b^–Eα_52-68_ peptide–MHCII complex.^21^ We demonstrated that Tsc1-deficient DCs had increased antigen processing (Figure 6a). Previous studies showed that autophagy was an important source of antigens for CD4^+^ T cells,^22^ and mTORC1 inhibited autophagy.^23^ In our study, the autophagy was comparable between WT and Tsc1-deficient DCs and could be further promoted by rapamycin, which suggest that other pathways are involved in the autophagy in DCs (Figure 6b). In addition, the expression of migration-associated molecules chemokine (C-C motif) receptor 3 (CCR3), CCR5, CCR6 and CCR7^24^ were comparable between WT DCs and Tsc1-deficient DCs (Figure 6c). Thus, the impaired function of Tsc1-deficient DCs could not be attributed to impaired antigen processing, autophagy or migration.

We detected a considerably higher rate of apoptosis in *ex vivo* Tsc1-deficient DCs than in WT DCs (Figure 6d), although the proliferation was enhanced (Figure 6e), suggesting that increased apoptosis was the causative reason for the loss of DCs *in vivo*. In the DC-OT-II T cell co-culture system, the apoptosis of Tsc1-deficient DCs was remarkably higher than that of the WT DCs (Figure 6f), suggesting that insufficient DCs would be one of the reasons for impaired antigen-specific T-cell responses. The magnitude and quality of T-cell response is directly proportional to the number of antigen-pulsed DCs that arrived at the draining lymph nodes (LNs).^25^ The defective induction of antigen-specific T-cell responses by Tsc1-deficient DCs may be due to the decreased number of antigen-carrying DCs from the site of the challenge to the draining LNs. To test this, we used a model of fluorescein isothiocyanate (FITC) skin painting to track the transportation of DCs from the skin to the LNs. FITC-painted *CD11c*^Cre^*Tsc1*^f/f^ mice contained decreased FITC^+^ DCs in the draining LNs at 48 h (Figure 6g). Furthermore, B6.SJL mice were injected i.v. with the same number of carboxyfluorescein diacetate succinimidyl ester (CFSE)-stained WT or Tsc1-deficient DCs. After 3 days, we found less CFSE^+^ Tsc1-deficient DCs in the recipients' spleen and LNs (Figure 6h). These results proved that the declined numbers of antigen-pulsed Tsc1-deficient DCs arriving at draining LNs led to impaired function of Tsc1-deficient DCs in inducing antigen-specific T-cell responses *in vivo*.

### Tsc1 inhibit DCs apoptosis via repressing mTORC1 and ROS-Bim pathways

**Figure 7:**
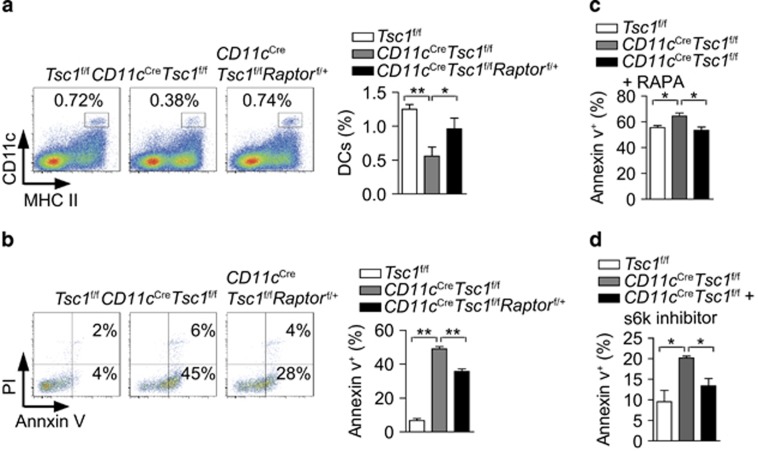
Tsc1 inhibits DCs apoptosis via repressing the mTORC1 pathway. (**a**) The percentage of DCs in the spleen from the indicated mice (*n*=6). (**b**) Annexin V and PI staining of splenic DCs from the indicated mice (*n*=3). (**c** and **d**) Apoptosis of splenic DCs, 16 h after i.v. injection with RAPA (30 *μ*g per mouse) or S6K inhibitor (50 *μ*g per mouse) (*n*=3). All mice analyzed were 6 week old. *P<0.05, **P<0.01; error bars represent S.D.; all data are representative of at least three independent experiments

We further explored the molecular mechanisms by which Tsc1 prevents DCs from undergoing apoptosis. Partially reduced activity of mTORC1 can rectify the reduction in the DCs' number and increased apoptosis of Tsc1-deficient DCs (Figures 7a and b). Administration of the mTORC1 suppressor RAPA or S6K inhibitor *in vivo* also partially restored cell survival (Figures 7c and d). These results indicate that Tsc1 promotes DC survival partly via mTORC1.

**Figure 8:**
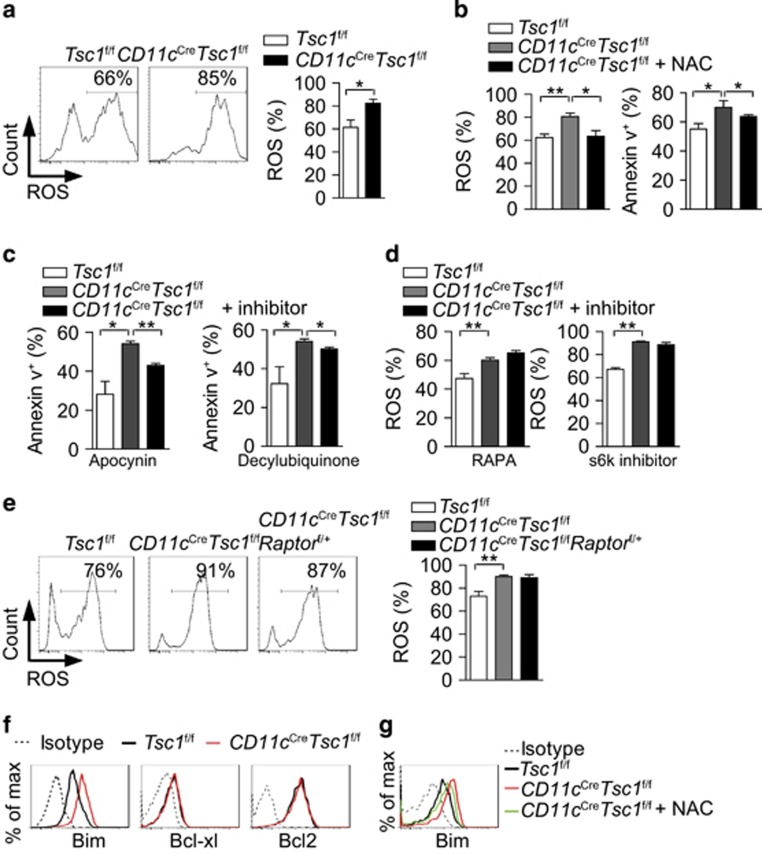
Tsc1 inhibits DCs apoptosis via repressing the ROS-Bim pathway. (**a**) ROS production of splenic DCs from the indicated mice (*n*=3). (**b**) ROS production and apoptosis of splenic DCs, treated with or without ROS scavenger NAC (5 mM) for 16 h (*n*=3). (**c**) Apoptosis of splenic DCs, treated with apocynin (10 *μ*M) or decylubiquinone (5 *μ*M) for 16 h (*n*=3). (**d**) ROS production of splenic DCs, 16 h after i.v. injection with or without RAPA (30 *μ*g per mouse) or s6k inhibitor (50 *μ*g per mouse) (*n*=3). (**e**) ROS production of splenic DCs from the indicated mice (*n*=3). (**f**) Expression of Bim, Bcl-xl, Bcl-2 in splenic DCs. (**g**) Expression of Bim in splenic DCs, treated with or without ROS scavenger NAC for 16 h. All mice analyzed were 6 week old. *P<0.05, **P<0.01; error bars represent S.D.; all data are representative of at least three independent experiments

ROS contributes to apoptotic cell death.^26^ We found that the level of ROS was increased in Tsc1-deficient splenic DCs (Figure 8a), which is consistent with the reported role of Tsc1 on ROS production in BM-DCs.^15^ The ROS scavenger *N*-acetyl-cysteine (NAC) alleviated the abnormal cell apoptosis (Figure 8b). NADPH oxidases (NOXs) and mitochondria are major source of endogenous ROS,^27^ and we found that both the NOX inhibitor (apocynin) and mitochondria respiration inhibitor (decylubiquinone) can rescue the apoptosis (Figure 8c). These results suggest that increased ROS production from NOX system and mitochondria may promote the apoptosis of Tsc1-deficient DCs. Surprisingly, administration of the mTOR suppressor RAPA or the S6K inhibitor *in vivo* did not suppress ROS levels in Tsc1-deficient DCs (Figure 8d) but partially restored cell survival (Figures 7c and d), which were also confirmed in *CD11c*^Cre^*Tsc1*^f/f^*Raptor*^f/+^ conditional mice (Figure 8e). These results indicate that mTORC1 signaling does not regulate ROS production in Tsc1-deleted DCs. Intracellular staining of the Bcl family of proteins revealed that the levels of pro-apoptotic protein Bim were elevated in Tsc1-deficient DCs, but the levels of antiapoptotic Bcl-xl and Bcl-2 were unaltered (Figure 8f). The ROS/JNK signaling pathway has been shown to upregulate Bim and lead to Bax-mediated apoptosis.^28^ Indeed, NAC reduced ROS production (Figure 8b) and Bim expression (Figure 8g), supporting the hypothesis that the ROS-Bim pathway is one of the major cause of apoptosis in Tsc1-deficient DCs. Taken together, Tsc1 promotes DC survival via the independently repression of the mTORC1 and ROS-Bim pathways.

## Discussion

The molecular mechanisms by which DCs regulate T-cell homeostasis in steady-state conditions and antigen-specific T-cell response remain incompletely understood. Recent publications demonstrate that Tsc1 regulates DC development and activation through mTORC1/Myc-dependent and -independent signals.^9, 15^ In contrast, our study highlights the role of Tsc1 in mature DCs that maintains naive T-cell quiescence in steady-state conditions but promotes antigen-specific T-cell responses.

In this paper, we demonstrated for the first time that Tsc1-deficient DCs resulted in the development of lymphoproliferative disorder, disturbed serum immune globulin and limited weight gain in mice, which indicates the role of Tsc1 in DCs in preserving immune tolerance. Selective loss of Tsc1 in DCs perturbs T-cell homeostasis and causes spontaneous activation of naive T cells, which were not observed previously. Pro-Inflammatory cytokines promote naive T-cell proliferation.^29^ Pan *et al.*^14^ indicated that Tsc1 deficiency increased the expression of TNF and IL-6 in BM-derived DC, which was not detected in splenic DCs in our study. Nrp1 expression by DCs has been reported to be essential for activation of naive T cells and thus the initiation of primary immune responses. Meanwhile, the expression of co-stimulatory molecules CD40, CD80 and CD86 was not significantly altered in splenic DCs, which was increased in BM-derived DCs.^15^ We found that Tsc1 deficiency led to mTORC1-PPAR*γ*-dependent upregulation of the expression of Nrp1 on DCs, which led to the aberrant proliferation of naive T cells. Therefore, in steady-state conditions Tsc1 functions as a tolerant factor repressing Nrp1 expression by DCs, thus avoiding aberrant immune activation. Several studies have suggested that Tsc1 mutation in the patients with human tuberous sclerosis complex have caused immune activation, which might further contribute to the development of the disease.^30, 31, 32, 33^ The spontaneous T-cell activation and lymphoproliferative disease in mice lacking Tsc1 in DCs implies that disruption of Tsc1-dependent DC function may be a mechanism underlying the immune activation phenotype in human tuberous sclerosis complex patients. Nevertheless, future studies are needed to address whether Tsc1 deficiency may cause susceptibility to autoimmune disorders or immunodeficiency disorders in tuberous sclerosis complex patients.

Despite their effect on promoting naive T-cell proliferation, Tsc1-deficient DCs failed to mount an efficient antigen-specific OT-I/OT-II T-cell immune response *in vivo* and *in vitro*. In contrast, Tsc1-deficient BM-derived DCs seemed to have a normal ability to activate MHC-I-restricted OT-I T cells *in vitro*.^14^ Efficient presentation of antigen to T cells results in activated antigen-specific T-cell response.^5^ Previous studies demonstrated that autophagy was an important source of antigens for CD4^+^ T cells^22^ and mTOCR1 functioned as a transcriptional regulator of autophagy.^23^ Here we showed that the ability of antigen presentation was elevated and the autophagy was unchanged in Tsc1-deficient DCs, ruling out their roles in impaired antigen-specific T-cell immune response. Although mTOCR1 signaling was activated in Tsc1-deficient DCs, the autophagy was unaltered, indicating that other signaling pathways regulate the autophagy in DCs. Distinct from the previous study with *in vitro*-cultured BM-derived DCs, Tsc1-deficient splenic DCs did not display significant decreased expression of IRF4/CIITA/MHC II and IL-12, limited proliferation or abnormal differentiation.^14, 15^ Instead, we found that, owing to remarkably impaired survival during antigen transportation and presentation, the ability of Tsc1-deficient DCs to prime antigen-specific T-cell response was severely compromised. These results indicate that *in vitro*-generated DCs are different from *in vivo* DCs and *in vitro* findings must be validated *in vivo*.^2^ Furthermore, Wang *et al.*^15^ reported that Tsc1-deficient BM-derived DCs had a defective ability to drive Th1 differentiation, nd our study has shown that the splenic DC subsets lacking Tsc1 exhibited a defective ability to drive Th2 and Th17 differentiation, the mechanisms of which need further elucidation.

Tsc1 promoted DCs' survival through repressing the mTORC1- and mTORC1-independent ROS-Bim pathways, respectively. Wang *et al.*^15^ showed that deletion of Tsc1 and Rheb, a small GTPase that activated mTORC1, in BM-derived DCs almost completely restored the increased apoptosis of Tsc1-deficient DCs, associated with the reversal of ROS overproduction and dysregulated expression of Bim,^8^ indicating that Tsc1/mTORC1 regulates ROS and Bim production. The simplest interpretation of diverse results is that the Tsc1 regulates distinct pathways in *in vitro*-generated DCs and *in vivo* DCs. In addition, Rheb also regulates mTORC1-independent pathways.^34^ These results suggested that Tsc1 in DCs preserves the antigen-specific T-cell response and the Tsc1 function and acting mechanisms in *in vivo* DCs were very different from that in *in vitro*-cultured BM-derived DCs.

Thus the immune system utilizes the Tsc1 enzyme to restrain mTORC1 activity and to preserve survival and function of DCs, ensuring normal immune homeostasis and function.

## Materials and Methods

### Mice

All animals were maintained in specific pathogen-free barrier facilities and were used in accordance with protocols approved by the Institutional Animal Care and User Committee at the Institute of Hematology, Chinese Academy of Medical Sciences. C57BL/6 (CD45.2) and B6.SJL (CD45.1) were provided by the Animal Centre of the Institute of Hematology and Hospital of Blood Diseases, Chinese Academy of Medical Sciences and Peking Union Medical College (Tianjin, China). *CD11c*^Cre^, *Tsc1*^f/f^, *Raptor*^f/f^, OT-I and OT-II mice were purchased from Jackson Laboratories (Bar Harbor, ME, USA). All used mice have been backcrossed with C57BL/6 mice for at least five generations. *Tsc1*^f/f^ mice were crossed with *CD11c*^Cre^ transgenic mice to generate *CD11c*^Cre^*Tsc1*^f/f^ and *Tsc1*^f/f^ mice (WT mice). *CD11c*^Cre^*Tsc1*^f/f^ mice were crossed with *Raptor*^f/f^ mice to generate *CD11c*^Cre^*Tsc1*^f/f^*Raptor*^f/+^ mice.

### Antibodies and reagents

The following antibodies for flow cytometry were from BD Biosciences (Heidelberg, Germany), eBioscience (San Diego, CA, USA), Biolegend (San Diego, CA, USA) and Invitrogen (Calsbad, CA, USA): PerCP-cy5.5-anti-CD4, PerCP-cy5.5-anti-IFN-*γ*, PerCP-cy5.5-anti- MHC II, Alexa Fluor 700-anti-CD44, PE-anti-CD8, PE-anti-CD209, PE-anti-Nrp1, PE-anti-B7-DC, PE-anti-B7-H1, PE-anti-B7-H2, PE-anti-B7-H3, PE-anti-Ox40L, PE-anti-Bim, PE-anti-Bcl-xl, PE-anti-Bcl2, PE-anti-Foxp3, PE-anti-CCR3, PE-anti-CCR5, PE-cy7-anti- MHC I, PE-cy7-anti-CCR6, PE-cy7-anti-CCR7, FITC-anti-IL-4, APC-anti-CD11c, APC-anti-CD19, APC-anti-F4/80, APC-anti-IL-5, APC-anti-IL-17, APC-anti-CD45.1, APC-anti-CD62L, APC-cy7-anti-CD45.2, APC-cy7-anti-Ly6c, FITC-anti-Ly6g, FITC-anti-Y-Ae, FITC-anti-Annexin V, FITC-anti-Ki67, PE-cy7-anti-CD40, PE-cy7-anti-80, and PE-cy7-anti-CD86. Intracellular staining antibodies of phospho-Erk1/2 (Thr 202/Tyr 204), phospho-Akt (Ser 473), phospho-JNK (Tyr 185), phospho-p38 MAPK (Thr 180/Tyr182), phospho-NF-*κ*B p65 (Ser 536) and phospho-S6K (Ser 235/Ser 236) were obtained from Cell Signaling Technology (Beverly, MA, USA). Propidium iodide solution (PI) and NAC were obtained from Sigma-Aldrich (St. Louis, MO, USA). RAPA, apocynin, decylubiquinone, mTORC1 inhibitor everolimus, PPAR-*γ* inhibitor T0070907, STAT3 inhibitor S3I-201, HIF inhibitor 2-methoxyestradiol, ERK inhibitor FR 180204, JNK inhibitor IX and S6K inhibitor PF-4708671 were obtained from Selleck (Houston, TX, USA).

### Cell purification, flow cytometry and intracellular staining

Single-cell suspensions were prepared from spleen and peripheral LNs for staining or cell purification. CD11c^+^ DCs were purified with CD11c MicroBeads (Miltenyi Biotec, Bergisch Gladbach, Germany). CD4^+^/CD8^+^ T cells were purified from the spleen and LNs of OT-II/OT-I transgenic mice by Dynabeads Untouched Mouse CD4/CD8 Cells Kits (Invitrogen). Cell sorting, flow cytometry and intracellular staining were performed as described.^35^ Flow cytometric data were acquired with LSR II (BD Biosciences) and were analyzed with the FlowJo7.6 software (TreeStar, Ashland, OR, USA). Gates were determined through the use of unstimulated control cells or isotype-matched control antibodies where appropriate. For intracellular staining of cytokine, cells were stimulated for 4 h *ex vivo* with phorbol myristate acetate (50 ng/ml, BD Biosciences) and ionomycin (500 ng/ml, BD Biosciences). Surface antigens on cells were stained, then fixed and made permeable with the Cytofix/Cytoperm Kit (BD Biosciences), and then intracellular cytokines in cells were stained for 1 h. For intracellular staining of phosphorylated proteins, cells were stimulated for 30 min *ex vivo* with or without lipopolysaccharides (LPS; Sigma-Aldrich). Surface antigens on cells were stained, cells were fixed with 2.0% formalin and permeabilized with methanol and phosphorylated proteins in cells were stained for 1 h. ROS production was determined by CM-H2DCFDA (C-6827, Thermo Fisher Scientific, Billerica, MA, USA).

### Immunoblot analysis

Sorted DCs were lysed and SDS-PAGE was performed as described.^35^ Antibodies to PPAR-*γ*, Bim and Actin were from Cell Signaling Technology.

### Enzyme-linked immunosorbent assay (ELISA)

Concentrations of secreted cytokines, serum immunoglobulins and auto-reactive antibodies were measured by commercial ELISA kit according to the manufacturer's instruction. Purified WT or Tsc1-deficient DCs (1 × 10^6^) were plated into each well of 48-well plates and treated with LPS (10 ng/ml) for 24 h. The culture supernatants were harvested to detect IL-1a, IL-6, IL-12a p40, IL-12a p70, TNF-α, IL-4, IL-5 and TGF-*β*1 cytokines (Biolegend). Blood (500 *μ*l) was taken from WT or *CD11c*^Cre^ *Tsc1*^f/f^ mice. The serum was separated and used to evaluate the concentrations of serum immunoglobulins IgG1, OVA- IgG1, IgE, IgM and IgA (R&D Systems, Minneapolis, MN, USA) or auto-reactive antibodies TSHR, ISR, ANA, ssDNA and C1q-A band UACA IgG (Mlbio, Shanghai, China). Measurements were made at 405 nm with Synergy 2 microplate reader (BioTek, Winooski, VT, USA).

### *In vitro* DC-mediated antigen-independent naive T-cell proliferation

Purified CD4^+^CD44^−^CD62L^+^ naive T cells were labeled with CFSE (2 *μ*M; Thermo Fisher Scientific) for 5 min at 37 °C and then washed twice with phosphate-buffered saline (PBS). In all, 5 × 10^4^ WT or Tsc1-deficient DCs were co-cultured with 1 × 10^5^ CFSE-stained T cells for 3 days in which the physiological T-cell survival factor IL-7 (100 ng/ml, PeproTech, Rocky Hill, NJ, USA) was added.^36^ CFSE profiles were examined by flow cytometry after culturing.

### *In vitro* and *in vivo* DC-mediated antigen-specific T-cell proliferation

Purified OT-I/OT-II T cells were labeled with CFSE (2 *μ*M) for 5 min at 37 °C and then washed twice with PBS. For *in vivo* studies, 3 × 10^6^ T cells were transferred into WT or *CD11c*^Cre^*Tsc1*^f/f^ mice by tail vein injection. After 1 day, mice were challenged by subcutaneous injection of 5 *μ*g OVA protein (Sigma-Aldrich) with 100 *μ*l complete Freund's adjuvant in each flank. After 3 days, inguinal LNs were removed and analyzed by flow cytometry. For *in vitro* studies, 5 × 10^4^ WT or Tsc1-deficient DCs were loaded with OVA_323-339_ or OVA_257-264_ (2 mg/ml, Sigma-Aldrich) overnight. Purified OT-I/OT-II T cells (1 × 10^5^) were labeled with CFSE and co-cultured with DCs in a 96-well plate for 3 days. CFSE profiles were examined by flow cytometry after culturing.

### Asthma model

Mice were sensitized as previously described.^37^ Six-to-8 week-old mice were injected intraperitoneally on days 0, 7 and 14 with 50 *μ*g/mouse of OVA (Sigma-Aldrich), emulsified in Al (OH)_3_. Mice were challenged inhalation of aerosolized OVA solution (25 *μ*g per mouse) on days 21, 22 and 23 and killed for analysis on day 25. Mice in the control group received mock sensitization with Al(OH)_3_ and were challenged with an aerosol of saline without OVA. Bronchoalveolar lavage fluid was prepared by washing the lungs three times with 0.5 ml ice-cold PBS. The cells were sedimented by centrifugation at 400 × *g* for 10 min at 4 °C.

### *In vitro* peptide–MHC class II complex formation assay

In all, 0, 25, or 100 *μ*g/ml peptide (RLEEFAKFASFEAQGALANIAVDKANLDVMKKR) was administered in culture for 6 h before DCs were collected for analysis.^21^ Processed Eα 52–68 peptides (underlined a.a. sequence) in complex with I-A^b^ were detected using an antibody that specifically recognizes this peptide–MHC class II complex (Y-Ae, eBioscience).

### The CYTO-ID autophagy detection

The CYTO-ID Autophagy Detection Kit (ENZO, New York, NY, USA) was used to detect the autophagy of isolated splenic DCs, according to the manufacturer's instruction. DCs were treated with or without rapamycin for 16 h. The samples were examined by flow cytometry.

### FITC painting

The skin of the scapular region of WT and *CD11c*^Cre^*Tsc1*^f/f^ mice was painted with 20 *μ*l of 1% FITC in carrier solution (acetone: dibutyl pathalate, 1:1).^38^ After 48 h, draining LNs were collected, stained and analyzed by flow cytometry.

### *In vivo* antigen transportation and presentation

Purified WT or Tsc1-deficient DCs (1 × 10^6^) were labeled with CFSE and injected i.v. into recipient B6.SJL (CD45.1) mice. After 3 days, splenocytes and LN cells from the recipients were stained and analyzed.

### Statistics

An unpaired two-tailed Student's *t*-test (for two-group comparisons) or a two-way ANOVA (for more than two-group comparisons) was performed by Prism (GraphPad, San Diego, CA, USA) to calculate the statistical significance. *P*-values of <0.05 were considered significant.
