# Supplementary material for: Tsc1 expression by dendritic cells is required to preserve T-cell homeostasis and response
Source: Cell Death Dis. 2017 Jan 12;8(1):e2553–. doi: 10.1038/cddis.2016.487 (PMC5386387; doi:10.1038/cddis.2016.487)
Supplement: Supplementary Figures [file cddis2016487x1.docx]

**Supplementary Information**

**Supplementary Figure 1. Tsc1 deficiency does not induce significant alterations of specific markers of** [**autoimmune**](javascript:void(0);) [**disease**](javascript:void(0);)**.** Blood were taken from WT or *CD11c*^Cre^*Tsc1*^f/f^ mice. The serum was separated and used to evaluate the concentrations of markers of [autoimmune](javascript:void(0);) [disease](javascript:void(0);), determined by ELISA. TSHR: thyroid stimulating hormone receptor; ISR: insulin receptor; ANA: anti-nuclear antibody; ssDNA: single stranded DNA; C1q-Ab: complement 1q antibody; UACA: uveal autoantigen with coiled-coil domains and ankyrin repeats. Error bars represent SD; all data are representative of at least three independent experiments.

**Supplementary Figure 2. Tsc1 sustains DCs quiescence and survive *in vivo*.** (**a**) Cell size and expression of indicated molecules on splenic DCs. (**b**) Concentration of splenic DCs cytokins. Purified splenic DCs were stimulated with LPS for 24 h. The culture supernatants were harvested and cytokines were determined by ELISA. (**c**) The percentage and absolute number of cDCs and pDCs in spleen (Spl) and lymph nodes (LNs) (n=5). All mice analyzed were 6-week-old. Error bars represent SD; all data are representative of at least three independent experiments.
